# Novel Applications of Magnetic Cell Sorting to Analyze Cell-Type Specific Gene and Protein Expression in the Central Nervous System

**DOI:** 10.1371/journal.pone.0150290

**Published:** 2016-02-26

**Authors:** Leanne Melissa Holt, Michelle Lynne Olsen

**Affiliations:** 1 Department of Cell, Developmental and Integrative Biology, University of Alabama at Birmingham, Birmingham, Alabama, United States of America; 2 Center for Glial Biology in Medicine, University of Alabama at Birmingham, Birmingham, Alabama, United States of America; The Ohio State University, UNITED STATES

## Abstract

The isolation and study of cell-specific populations in the central nervous system (CNS) has gained significant interest in the neuroscience community. The ability to examine cell-specific gene and protein expression patterns in healthy and pathological tissue is critical for our understanding of CNS function. Several techniques currently exist to isolate cell-specific populations, each having their own inherent advantages and shortcomings. Isolation of distinct cell populations using magnetic sorting is a technique which has been available for nearly 3 decades, although rarely used in adult whole CNS tissue homogenate. In the current study we demonstrate that distinct cell populations can be isolated in rodents from early postnatal development through adulthood. We found this technique to be amendable to customization using commercially available membrane-targeted antibodies, allowing for cell-specific isolation across development and animal species. This technique yields RNA which can be utilized for downstream applications—including quantitative PCR and RNA sequencing—at relatively low cost and without the need for specialized equipment or fluorescently labeled cells. Adding to its utility, we demonstrate that cells can be isolated largely intact, retaining their processes, enabling analysis of extrasomatic proteins. We propose that magnetic cell sorting will prove to be a highly useful technique for the examination of cell specific CNS populations.

## Introduction

Recent research highlights the need to study cell populations in isolation to determine cell-type specific gene and protein expression patterns [1–8]. This is a considerable challenge in the central nervous system (CNS) where multiple cell types including neurons, astrocytes, oligodendrocytes, and microglia are densely packed. This challenge is exacerbated by the complex morphology of neural cells, which typically extend many long filamentous processes throughout the brain parenchyma and associate intimately with one another. Furthermore, excitotoxic mechanisms—which contribute to cellular damage and cell death—occur upon tissue disruption and are unavoidable during cellular dissociation. Despite these obstacles, several techniques have been used successfully to isolate or enrich different CNS populations, including immunopanning [9–11], percoll density gradient centrifugations [12, 13], laser capture micro-dissection (LCM) [5, 6, 12], fluorescent-activated cell (FAC) sorting [13–17], and the use of magnetically labeled antibodies to target specific cell types [7, 18, 19].

In adult CNS, FACs and LCM are the techniques of choice to separate cell types, each with their own inherent advantages and disadvantages. FAC sorting allows the separation and capture of cells using fluorescently-tagged antibodies, which are cell type specific. Alternatively, fluorescent reporters driven by cell type specific promoters are a common way of labeling and identifying a cell type of interest [15–17]. However, during the process of FACs, cells are carried in a stream of solution at relatively high velocity, shearing off complex CNS cellular processes and limiting the utility of this technique when extrasomatic proteins are being investigated. In contrast, LCM enables the user to trace the cell of interest, allowing cell bodies and their processes to be ‘captured’ [6, 12]. LCM is dependent on morphological assessment, which may be difficult to distinguish for some cell types or too subjective a measure [12]. Although highly specific, LCM is a low throughput method requiring considerable researcher time. Both FACS and LCM require costly, specialized equipment that necessitates training and may not be readily available to all researchers.

The isolation of cell populations using magnetically labeled antibodies targeted to cell-type specific surface antigens is a technique that has been available for nearly thirty years [19]. Traditionally utilized to isolate cell populations for *in vitro* analysis, [18, 20] more recent publications demonstrate that this technique can successfully purify CNS cell types in rodents at early postnatal ages (<postnatal day 7) [5, 21]. A major drawback to this method has been the inability to isolate enriched populations in the CNS in adult animals, which greatly limits its utility. Here, we demonstrate for the first time that magnetic cell sorting successfully sorts neuronal, astrocytic, and microglia cell populations in adult rodent brain. In addition, the sorting can be customized, enabling isolation of cell populations not only across development but also across species. RNA, which can be utilized for downstream applications including quantitative PCR and RNA sequencing, is obtained at relatively low cost without the need for specialized equipment or fluorescently labeled cells. This technique is inexpensive and efficient, minimizing critical time from brain harvest and cell dissociation to “cell capture” (approximately 1 hour to 90 minutes). Adding to its utility, we demonstrate that this technique is gentler than FAC sorting, allowing for the retention of cell processes and enabling analysis of membranous proteins.

## Materials and Methods

### Animals

All experimental protocols were in accordance with the NIH guidelines and were carried out with approval from the Animal Care and Use Committee of the University of Alabama at Birmingham (Permit Number: 09409). All animals were maintained on a 12 hour light/dark cycle with food and water available *ad libitum*. Every effort was made to minimize pain and discomfort. Wildtype C57/B6 mice and wildtype S100 GFP rats were used for these experiments.

### Dissection and Dissociation

Animals (mice and rats) were anesthetized and decapitated. Cortices were micro-dissected and meninges removed in ice cold ACSF (120mM NaCl, 3.0 mM KCl, 2mM MgCl, 0.2 mM CaCl, 26.2 mM NaHCO3, 11.1 mM glucose, 5.0 mM HEPES) bubbled with 95% oxygen. To reduce neuronal cell death and excitotoxicity, AP5 (3 mM) and CNQX (3 mM) were added to the ACSF [22]. We found that one cortical hemisphere provided sufficient material at all ages evaluated. Experiments were performed with as little as 0.06 grams of tissue, equivalent to one-half of a p7 (postnatal day 7) cortex, through 0.20 grams of tissue (one entire cortex of adult-p80). Following cortical isolation, the tissue was minced into 1 mm^3^ pieces and was dissociated using Worthington’s Papain Dissociation kit (catalog number LK003153) with the following modifications: 1) tissue was left in dissociation medium for 15–25 minutes and 2) oxygen was continuously perfused over (not bubbled within) the solution for the duration of the incubation period. The duration of dissection and dissociation took approximately 25 minutes. Following dissociation, an aliquot of the suspension was removed to use as matched whole cortex controls. Astrocytes, neurons, or microglia were isolated as described below. Information on kits and antibodies utilized to isolate cellular populations has been summarized in S1 Table.

### Microglia/myelin debris removal and microglial isolation

Prior to isolation of astrocyte or neuronal cellular populations, we performed a debris removal step using modified protocols from Miltenyi Biotec’s Myelin Removal Kit (catalog number 130096733) and Cd11b^+^ Microbeads (catalog number 130093634). Following dissociation, up to 10^7^ cells were suspended in 200 μL 0.5% BSA in PBS buffer and incubated concurrently with 20 μL anti-myelin microbeads and 20 μL anti-Cd11b^+^ microbeads for 15 minutes at 4°C for subsequent astrocyte population isolation, or with anti-myelin microbeads alone for subsequent neuronal population isolation. Cells were then washed with 1mL 0.5% BSA in PBS buffer and centrifuged at 300x*g* for 5 minutes to remove any unbound beads from the pellet. After disposal of the wash solution, the pellet was resuspended in either 500 μL buffer for further astrocyte processing or 1 mL buffer if neuronal processing was desired, and the suspension added to a prepped LS column fitted in MACSMidi magnetic cell separator, with collection of the flow through. The column was further washed three times with 3mL buffer to ensure removal of unlabeled cells. The cells retained on the column, which largely represent oligodendrocyte and microglia cell populations, were eluted in 5mL buffer. The flow through was used in subsequent steps to isolate either astrocyte or neuronal populations.

Specific isolation of the microglial population for further protein and RNA processing was also performed prior to customized astrocyte isolation. For this process, dissociated cells were initially suspended in 90uL buffer and incubated with Cd11b^+^ microbeads alone, followed by the above-described steps outlined for subsequent astrocyte isolation. After collection of flow through, microglia expressing Cd11b were eluted from the column using 5mL buffer, and RNA and protein extraction followed.

### Isolation of ACSA-2-expressing cells

**Fig 1:**
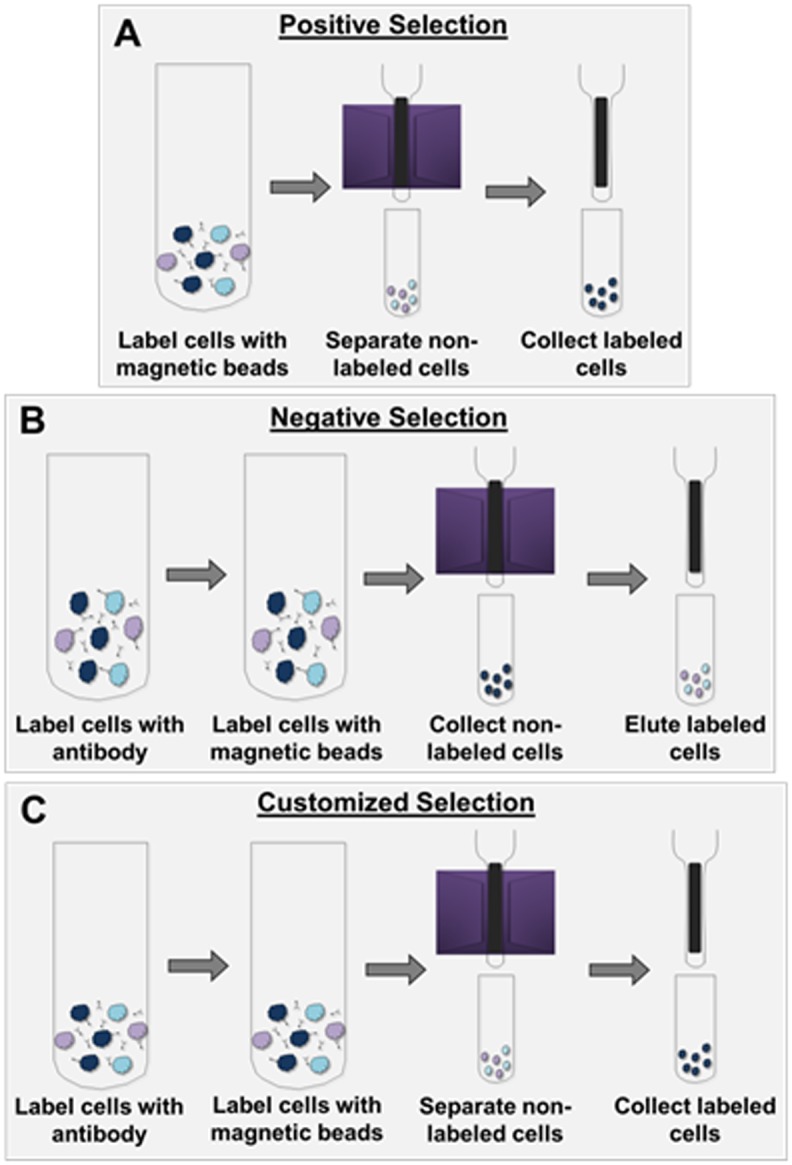
Schematic of cell separation techniques. A) Positive selection of astrocytes was performed using an ACSA-2 antibody. Dissociated cells were incubated with magnetic beads conjugated with an ACSA-2 antibody, and were applied to a ferromagnetic column placed in a magnetic field. The astrocyte enriched cell population was eluted from the column after successive washes that removed non-target cell populations. B) Neurons were enriched using a negative selection protocol. Dissociated cells were incubated with biotin-conjugated antibodies targeting non-neuronal cell populations, followed by labelling with streptavidin-coated magnetic beads. Neuronal cell populations were eluted, while biotin-bound cells were retained on the magnetic column. C) Positive selection of cells is customizable. Here an antibody which detects an extracellular epitope of GLT-1 was used to enrich astrocytes. Dissociated cells were labeled with the rabbit-anti-GLT-1 antibody and subsequently labeled with anti-rabbit microbeads. The enriched population was retained on the column, and eluted subsequent to washing undesired cells from the magnetic column.

Using a modified protocol from Miltenyi Biotec’s Anti-ACSA-2 kit (catalog number 130097678), astrocytes were positively selected (Fig 1A). ACSA-2 (astrocyte specific cell surface antigen 2) was developed by Miltenyi to detect astrocytes in a CNS homogenate and is specific to astrocytes. As detailed in the results section, we have indeed confirmed that ACSA2^+^ cells express astrocytic mRNA and protein and lack expression of RNA or protein specific for other CNS cell populations. Up to 10^7^ dissociated cells were suspended in 150 μL 0.5% BSA in PBS buffer and incubated with 20 μL FcR Blocking Buffer for 25 minutes at 4°C, followed by incubation with 20 μL ACSA-2 MicroBeads for 25 minutes at 4°C. Cells were then washed with 1mL 0.5% BSA in PBS buffer and centrifuged at 300x*g* for 5 minutes to remove excess beads from the solution. Following disposal of the wash solution and according to the manufacturer’s guidelines of maximum column capacity, the pellet was resuspended with 500μL buffer, and the suspension added to a prepped LS column fitted in MACSMidi magnetic cell separator. The column was washed with 3mL buffer three successive times to remove unlabeled cells. Following column removal from the magnetic separator, astrocytes were eluted in 5mL buffer. Cell number was then determined and total RNA or protein was extracted.

### Neuronal isolation

Isolation of neuronal cell populations was similarly performed following both tissue dissociation and the above-described myelin debris removal step. Neurons were negatively selected using Miltenyi Biotec’s Neuronal Isolation Kit (catalog number 130098752) (Fig 1B). Following a modified protocol, up to 10^7^ dissociated cells were resuspended in 150μL 0.5% BSA in PBS buffer and incubated with 20μL biotinylated antibodies for 10 minutes at 4°C. Cells were washed with 1mL buffer and centrifuged at 300x*g* for 5 minutes, which allowed unbound antibody to remain in solution, separate from the cellular pellet. Following disposal of the wash buffer, cells were resuspended in 150μL 0.5% BSA in PBS buffer and incubated with 20μL streptavidin coated microbeads for 15 minutes at 4°C. Following the manufacturer’s guidelines of maximum column capacity, the suspension was brought to 500μL with buffer, and added to a prepped LD column fitted in a MACSMidi magnetic cell separator. Neurons were depleted with two 1mL washes. Unwanted, labeled cells were eluted with 3mL buffer. Cell numbers were then determined and total RNA or protein was extracted.

### Isolation of Glt-1 expressing cells

Astrocytes expressing Glt-1 were positively selected using Alomone’s extracellular rabbit anti Glt-1 antibody (catalog number AGC-022, Fig 1C). Glt-1, a Na^+^-dependent glutamate transporter, is localized to mature astrocytes throughout the CNS. Following dissociation, up to 10^7^ dissociated cells were suspended in 150μL 0.5% BSA in PBS buffer and incubated with 20μL antibody for 30 minutes at 4°C. Cells were washed with 1-2mL buffer and centrifuged at 300x*g* for 5 minutes, which allowed unbound antibody to remain in solution, separate from the cellular pellet. After wash buffer disposal, cells were re-suspended in 150μL 0.5% BSA in PBS buffer and incubated with 20μL Miltenyi Biotec’s Anti-Rabbit Microbeads (catalog number 130048602) for 30 minutes at 4°C, followed by a 1mL wash. The suspension was centrifuged at 300x*g* for 5 minutes to help eliminate unbound beads from the pellet. After wash buffer disposal and per the manufacturer’s guidelines of maximum column capacity, cells were suspended in 500μL 0.5% BSA in PBS and applied to a prepped LS column fitted in MACSMidi magnetic cell separator. Non-labeled cells were collected with three washes of 3mL buffer. Labeled astrocytes expressing Glt-1 were eluted in 5mL buffer following column removal from the separator. Cell number was determined and RNA or protein was extracted.

### FAC Sorting

Following dissociation, as described previously, astrocytes were isolated by FAC sorting [23, 24]. Cells were sorted by using a FACS ARIAII Special Order Product (SORP). S100β eGFP was excited at 488nm and filtered with 505LP paired with a 530/30 BP filter. Previous experiments showed that cells high on the SSC axis had low RNA quality and yield, and were excluded (S1 Fig) [23]. Cells with low SSC were further sorted on GFP intensity.

### RNA Isolation and RT-PCR

**Fig 2:**
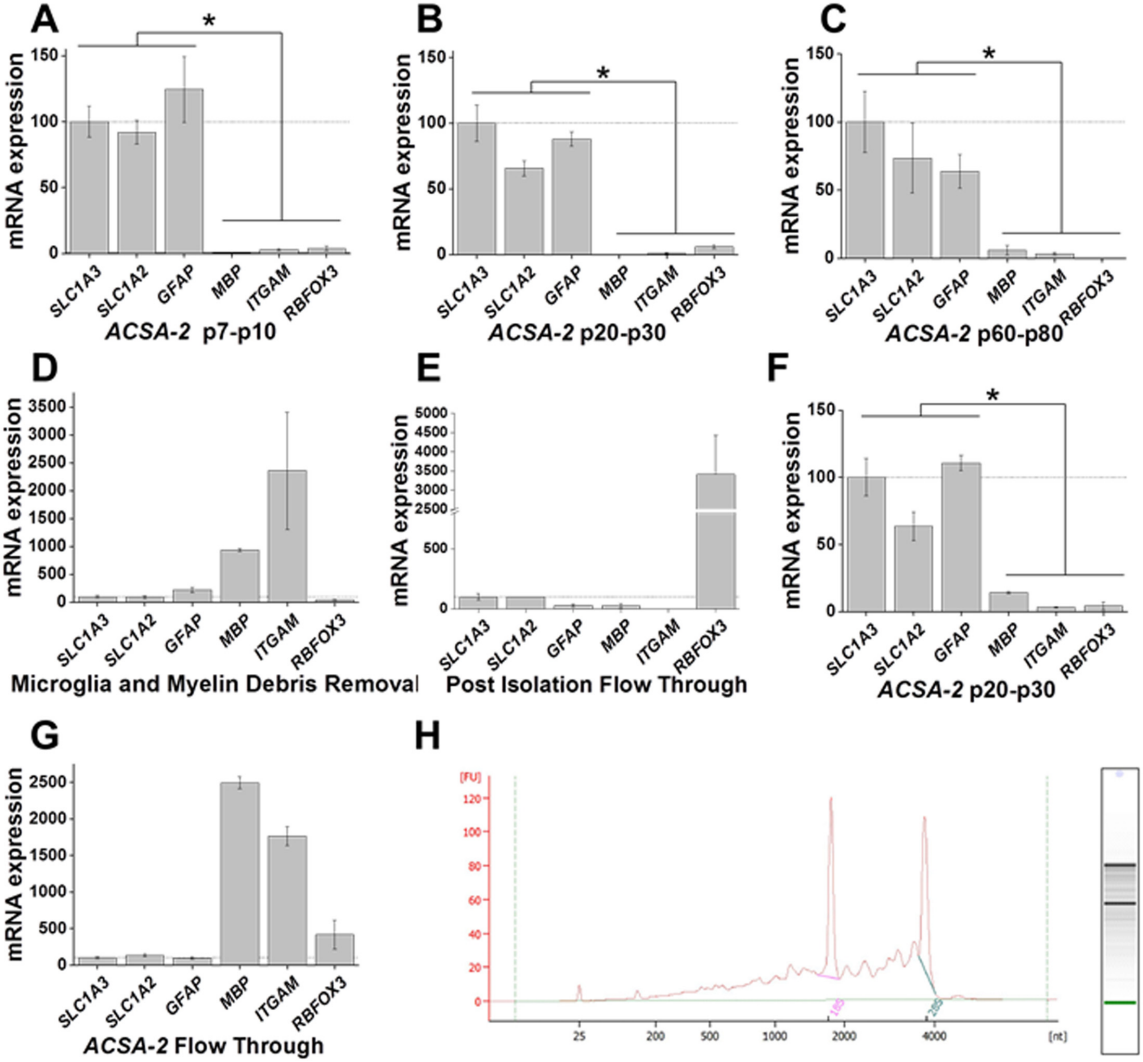
Isolation of astrocytes across development. Astrocytes were isolated using an ACSA-2 antibody conjugated to magnetic microbeads. A, B, C) Quantitative PCR data analysis of isolated astrocytes as demonstrated by expression of the astrocytic genes *SLC1A3*, *SLC1A2*, and *GFAP* in young (p7-p10), juvenile (p20-p30), and adult (p60-p80) mice. In contrast, there are low levels of expression of oligodendrocyte gene *MBP*, microglia gene *ITGAM*, and neuronal gene *RBFOX3*. D, E) The efficiency of the ACSA-2 positive selection technique is indicated by qPCR data which demonstrates depletion of astrocytic genes *SLC1A3* and *SLC1A2* in the microglia and myelin debris removal fraction as well as the non-targeted cell population or “flow through” (example shown from p20-p30 isolation). F) Quantitative PCR data analysis of isolated astrocytes without addition of “clean up” step demonstrates minimal contamination of microglia and oligodendrocyte genes, *ITGAM* and *MBP*, respectively (example shown from p20-p30 isolation). G) Quantitative PCR data analysis of flow through fraction from experiments omitting the microglia and myelin debris removal step demonstrates low expression of astrocyte gene expression. H) Chromatograph demonstrating RNA integrity isolated from enriched population. RNA isolated from an adult animal (p70s) was Dnase treated and analyzed for quality. For all time points qPCR data is represented as mean +/- SEM with a minimum of n = 3. The mean differences between relative gene expression were analyzed by ANOVA, *p < 0.05.

**Fig 3:**
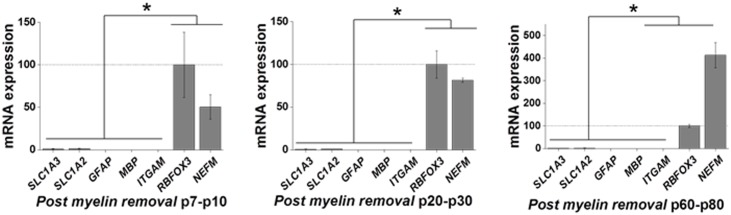
Isolation of neurons across development. A, B, C) Quantitative PCR analysis demonstrates isolation of neurons in young (p7-p10), juvenile (p20-p30), and adult (p60-p80) mice. Low expression of astrocytic gene *SLC1A3*, microglial gene *ITGAM*, and oligodendrocyte gene *MBP* was found with concomitant high expression of neuronal genes *RBFOX3* and *NEFM*. Data from qPCR is represented as mean +/- SEM with a minimum of n = 3 for all time points. The mean differences between relative gene expression were analyzed by ANOVA, *p < 0.05.

**Fig 4:**
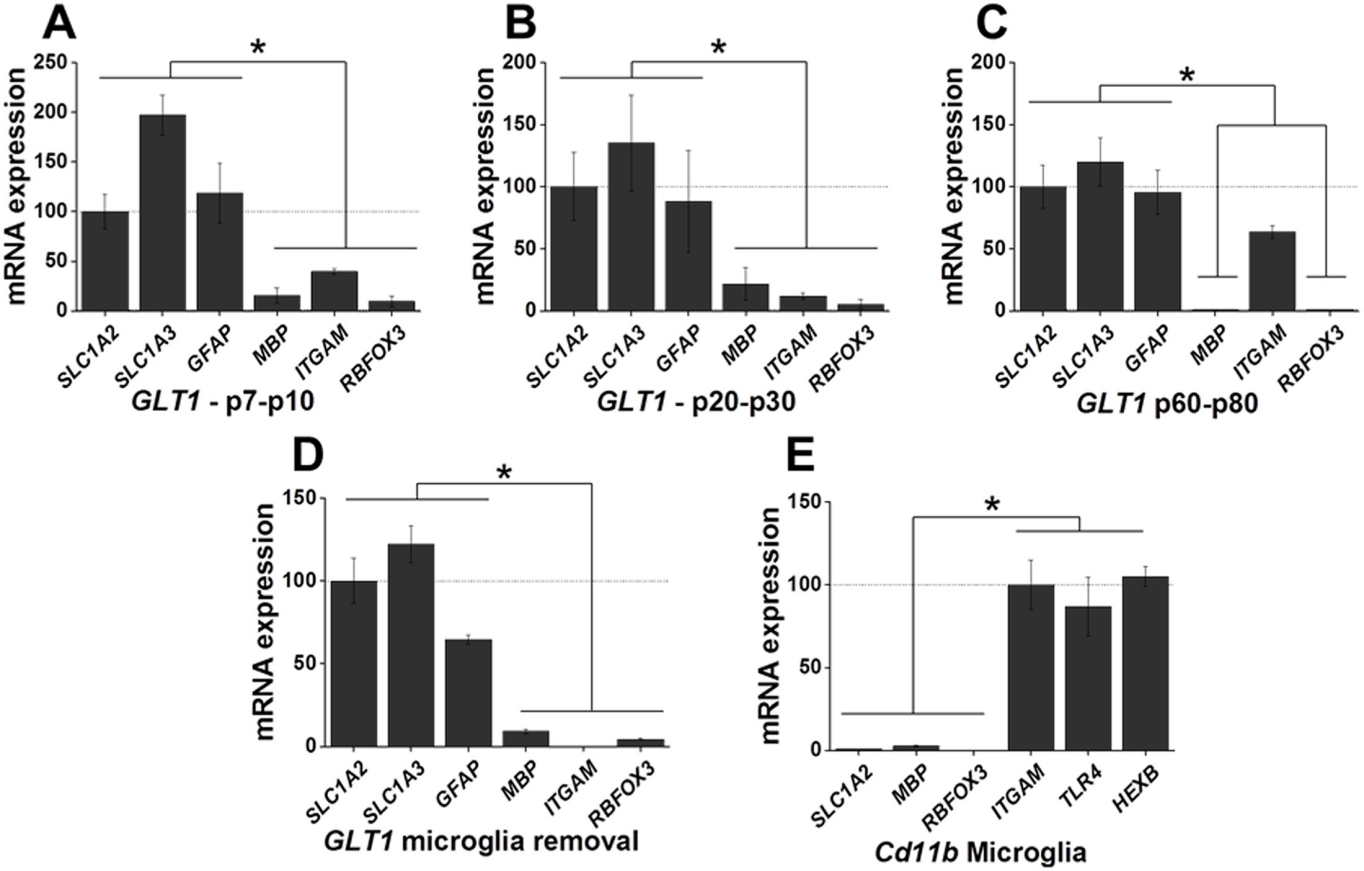
Customized isolation of astrocytes and microglia. Astrocytes were enriched using an antibody which recognizes an extracellular epitope of glutamate transporter GLT-1. A, B, C) Quantitative PCR analysis of the enriched astrocyte population in young (p7-10), juvenile (p20-p30), and adult (p60-p80) mice demonstrates enrichment of astrocytic genes *SLC1A3*, *SLC1A2*, and *GFAP* with low expression of oligodendrocyte and neuronal genes. C) Microglia contamination was seen in isolated populations from adult (p60-80) mice as indicated by moderate expression of microglia gene *ITGAM*. D) The microglia contamination was resolved by removing microglia with Cd11b+ microbeads prior to isolating astrocytes (example shown from p60-p80 isolation), as demonstrated by the minimal oligodendrocyte, microglia, and neuronal gene expression. E) Concomitantly, the microglia removal step allowed for the enrichment of Cd11b+ microglia in adult (p60) animals. Low expression of astrocytic, oligodendrocyte, and neuronal genes was found in the Cd11b+ isolated fraction. Data from qPCR is represented as mean +/- SEM with a minimum of n = 3 for all experiments. The mean differences between relative gene expression were analyzed by ANOVA, *p < 0.05.

**Fig 5:**
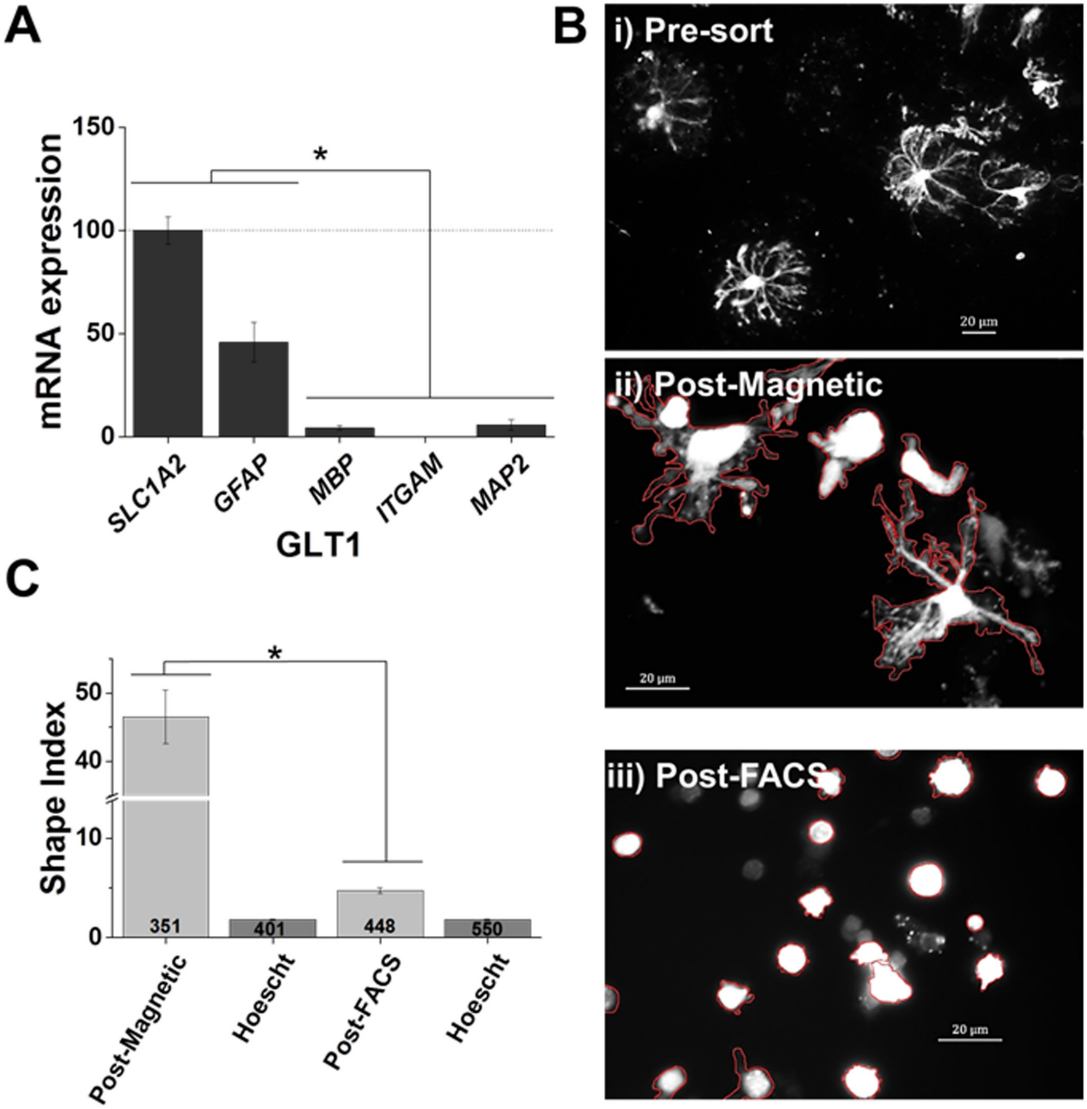
Increased retention of processes with magnetic separation. A) Analysis of the positive selection of astrocytes from rat cortex demonstrates a depletion of oligodendrocyte, microglia, and neuronal marker genes (*MBP*, *ITGAM*, *and MAP2*, respectively) with high expression of astrocytic gene expression, *SL1CA2* and *GFAP*, n = 6. The mean differences between relative gene expression were analyzed by ANOVA, * *p* < 0.05. B) Representative images of S100β eGFP positive astrocytes from i) pre-sorting, ii) post-magnetic sorting, and iii) post-FAC sorting of rat cortex. Images demonstrate that magnetic sorting resulted in an increased number of cells retaining complexity post-sorting. C) Shape Index quantification of cellular complexity demonstrates that magnetic sorting leads to an increased cellular complexity over FAC sorted cells. Mean Shape Index differences were analyzed by student’s *t*-test, *p < 0.001

Total RNA was isolated using Ambion’s PureLink RNA Mini Isolation kit and DNase treated (Invitrogen’s DNase I kit) according to the manufacturer’s instructions. Subsequently, 2 ng of RNA was reverse transcribed into cDNA using BioRad’s iScript kit and cDNA was normalized. The relative mRNA expression level was determined using real-time quantitative PCR by General Taqman PCR master mix. Relative mRNA expression levels were determined by the ddCt method, and normalized to matched whole cortex values to determine enrichment from baseline levels. As some primers used are developmentally regulated in expression, we then normalized values to a primer known to be a specific cell-type marker. For astrocytes isolated using ACSA-2, the gene *SLC1A3* was chosen (Fig 2A–2G); for neuron isolations, the gene *RBFOX3* was chosen (Fig 3A–3C); for astrocytes isolated using GLT-1, the gene *SLC1A2* was chosen (Fig 4A–4D and Fig 5A); for microglia isolations, the gene *ITGAM* was chosen (Fig 4E). Gapdh was used as an endogenous control for the normalization of RNA quantity. Primers used, their respective proteins, cell type expression, and localization are given in Table 1. RNA was DNase treated and quality analyzed by BioRad Experion Agilent 2100 Bioanalyzer.

**Table 1:** Genes and proteins used to distinguish cellular populations.

Gene	Protein	Cell-Type	Localization
*GFAP*	Gfap	Astrocytes	Cytoskeletal
*S100β*	S100β	Astrocytes	Cytosolic
*SLC1A2*	Glt-1	Astrocytes	Membrane
*SLC1A3*	Glast	Astrocytes	Membrane
*MBP*	Mbp	Oligodendrocytes	Membrane
*ITGAM*	Cd11b	Microglia	Membrane
*HEXB*	Hexb	Microglia	Lysosome
*TLR4*	Tlr4	Microglia	Membrane
*MAP2*	Map2	Neurons	Cytoskeletal
*NEFM*	Neurofilament	Neurons	Cytoskeletal
*RBFOX3*	NeuN	Neurons	Nuclear

### Immunofluorescent imaging and cellular complexity

Following dissociation, a small homogenate of the cell suspension was collected to image cells before further processing. Immediately following FAC sorting or collection of magnetic separation, cell suspensions were incubated in 1 mg/mL Hoechst for 5 minutes. Cell suspensions were then fixed by 4% paraformaldehyde for 15 minutes, resuspended in fresh PBS, and allowed to settle before imaging. Fluorescent images of eGFP+ cells were acquired with an Olympus VS-120 system.

Cellular complexity of isolated cells was determined by utilizing the Shape Index, given as $\frac{Perimeter^{2}}{area}-4\pi$ [25]. Area and perimeter of eGFP+/Hoechst+ cells were determined using ImageJ (1.48v) software.

### Protein extraction and Immunoblotting

Proteins were extracted by homogenizing samples in lysis buffer (1% sodium dodecyl sulfate (SDS), 100 mM Tris(hydroxymethyl)aminomethane (Tris) buffer, pH 7.5, supplemented with protease and phosphatase inhibitors (Sigma)), followed by two rounds of sonication for seven seconds. Lysates were centrifuged for 5 minutes at 16,000x*g*, followed by ThermoScientific’s Pierce BCA assay to determine protein concentration.

Proteins were heated to 60°C for 15 minutes with 2x loading buffer (100 mM Tris, pH 6.8, 4% SDS, in Laemmli-sodium dodecyl sulfate, 600 mM B-mercaptoethanol, 200 mM Dithiothreitol (DTT), and 20% glycerol). Equal amounts of protein per sample were loaded on a 4–20% gradient precast SDS polyacrylamide gel, followed by transfer to a PVDF membrane at 100V for one hour. Primary antibodies used were as follows: Aquaporin 4 (Alomone, rabbit, polyclonal, 1:2000); Gapdh (Millipore, chicken, polyclonal, 1:2000); Gfap (Millipore, mouse, polyclonal, 1:30,000); Glast (Abcam, rabbit, polyclonal, 1:500); and Glt-1 (Millipore, chicken, polyclonal, 1:10,000); Cd11b (Alamone, rabbit, polyclonal, 1:500); Map2 (Sigma, mouse, monoclonal, 1:500); NeuN (Millipore, rabbit, polyclonal, 1:500). All HRP secondary antibodies were used at a 1:2,000 concentration.

### Statistical Analyses

To determine statistical significance of enrichment in relative gene expression, values from quantitative PCR were utilized and pooled into cell-type specific categories. One-way ANOVAs followed by Tukey’s post-hot test were performed. A student’s t test was used when only a single comparison was made. Levene’s test of homogeneity was performed for all tests to ensure data followed a normal distribution. All data is represented as mean +/- SEM, with a minimum of n = 3 unless specified otherwise. Origin 9.1 statistical software was used for ANOVA statistical tests while t-tests were performed with Excel.

## Results

### Isolation of enriched cell populations across development

Magnetic cell separation has been available since the 1980s. In the CNS specifically, magnetic isolation has largely been utilized to isolate cells from early postnatal tissue for primary cultures, minimizing its utility for many research applications. We set out to determine if magnetic isolation could be used to reliably isolate different cell populations from CNS tissue across developmental time points from commercially available kits. We found that with minimal optimization, astrocytic, microglial, and neuronal populations were successfully enriched from a whole brain homogenate.

For these experiments, the genes *SLC1A3*, *SLC1A2*, *and GFAP* were used to identify astrocytes; *MBP* was used as an oligodendrocyte marker; *ITGAM*, *HEXB*, and *TLR4* were used to identify microglia; and *RBFOX3* and *NEFM* were used to identify neuronal cell populations. These genes, their respective protein products, cell type specific expression patterns, and cellular localization are listed in Table 1. In all experiments, a minimum of three independent experiments are shown.

Using the Anti-ACSA-2 MicroBead (ACSA-2) isolation kit for the isolation of astrocytes, we isolated ACSA-2 expressing cells from mouse cortex. Once dissociated and debris removed (see methods), cells were incubated with anti-ACSA-2 microbeads and added to a ferromagnetic column. Antibody-bound cells were retained on the magnetic column while unlabeled cells were removed with successive washes (Fig 1A). Quantitative PCR demonstrates a statistically significant enrichment of astrocytic genes and a concomitant depletion of oligodendrocyte, microglial, and neuronal gene expression relative to whole cortex from cortices dissociated at days p7-p10 (n = 3, p < 0.05) (Fig 2A). Similar results were obtained from cortical tissue dissociated from juvenile animals at p20-p30 (n = 3, p < 0.001) (Fig 2B). Performing the same experiment in adult mice cortices (p60-p80), we again observed an enrichment of astrocytic genes and depletion of gene expression from other CNS cell types (n = 3, p < 0.001) (Fig 2C). The efficiency of this positive selection method for the cell type of interest is demonstrated in Fig 2D and 2E where q-PCR data demonstrates a near depletion of astrocyte markers in both the debris removal fraction (Fig 2D) and the unbound eluate (Fig 2E). Data from one developmental point, p20-p30, is shown in Fig 2D and 2E. For comparison, we performed an astrocyte isolation from the P20-30 age group omitting the myelin debris and microglial removal (Fig 2F), as this step adds an additional 20–25 minutes. This resulted in slightly higher levels of other cell gene expression in the enriched astrocyte population. Additionally, qPCR data from the unbound eluate demonstrates near depletion of astrocyte gene expression (Fig 2G). Analysis of RNA indicated that the average RNA Integrity Number (RIN) of isolated astrocytes was not statistically different than that of whole cortex (p = 0.925) (S1 Table). A representative chromatograph of RNA isolated from astrocytes indicates primary bands at 18S and 28S with little RNA degradation (Fig 2H).

In the next set of experiments, we used an optimized protocol as detailed above and Miltenyi’s Neuronal Isolation kit to enrich neuronal cell populations. Dissociated cells from a cortex were incubated with biotinylated antibodies against non-neuronal antibodies supplied in the kit. The biotin-conjugated cells were retained on the column via biotin-streptavidin interaction, while the unlabeled neuronal cells were eluted off of the column (Fig 1B). Three independent experiments were performed for each developmental time point. The genes *GFAP*, *SLC1A2* and *SLC1A3* were used to identify astrocytes, *ITGAM* was used to identify microglia, and *MBP* was used to identify oligodendrocytes. Neuronal populations were identified using the genes *RBFOX3* and *NEFM* (Table 1). In the initial neuronal enrichment experiments, we observed significant expression of the oligodendrocyte gene *MBP*. This was attributed to a failure of oligodendrocytes to separate from the neurons as they pass through the column. As previous reports using fluorescent activated cell sorting to isolate neurons reported a myelin removal step prior to sorting to eliminate oligodendrocyte contamination [7, 15], we incorporated a myelin-removal step to the neuronal enrichment. Following removal of myelin, the oligodendrocyte contamination was resolved, leaving a relatively pure neuronal population. Neurons were successfully enriched at early postnatal (Fig 3A), juvenile (Fig 3B) and adult (Fig 3C) time points as indicated by the low expression levels of astrocyte, oligodendrocyte and microglial gene expression relative to the neuronal markers *RBFOX3* and *NEFM* (n = 3, p < 0.05).

### Magnetic enrichment can be customized

To determine if this technique was amenable to researcher customization, we next targeted astrocytes using Alomone’s commercially available extracellular-targeted GLT-1 antibody. GLT-1 is a membrane-targeted, Na^+^-dependent glutamate transporter, which is highly expressed in mature astrocytes. We chose this antibody because it recognizes an extracellular epitope of GLT-1, and is cross-reactive to multiple species, including mouse and rat. Using a positive selection method, mouse cortex was dissociated as previously described, and the resulting homogenate was incubated with the GLT-1 antibody and subsequently labeled with magnetic microbeads (Fig 1C). Cells bound to antibody/bead were retained on the column while unlabeled cells were washed through the column. As before, the genes *SLC1A3*, *SLC1A2*, and *GFAP* were used to identify astrocytes, *MBP* was used to identify oligodendrocytes, *ITGAM* was used to identify microglia, and *RBFOX3* was used to identify neuronal populations (Table 1). Quantitative PCR data demonstrates an enrichment of astrocytic genes with depletion of neuronal and oligodendrocyte marker genes in young (Fig 4A), juvenile (Fig 4B), and adult (Fig 4C) animals (p < 0.05). However, we did find that these experiments resulted in moderate levels of microglial gene expression in adult populations. This was attributed to an upregulation of GLT-1 by microglia, leading to some contamination [26, 27].

To resolve the microglia contamination, we utilized Miltenyi BioTec’s anti-Cd11b MicroBead kit. Following dissociation, cells were incubated with Cd11b^+^ microbeads and cells bound to the microbeads were retained on the ferromagnetic column. The unbound cell population was then used to further isolate GLT-1 positive astrocytes. This step added only an additional 15 minutes to the astrocyte isolation protocol. The adult (p60) time point was chosen for the following experiment, as it contained the highest levels of *ITGAM* expression and contamination. As seen in Fig 4D, qPCR data demonstrate that astrocytes were positively selected with high expression of astrocytic genes and concomitant low expression of oligodendrocyte, microglia, and neuronal genes, resolving the issue of microglia contamination in astrocytes isolated using the GLT-1 antibody (n = 3, p < 0.05). Furthermore, we were able to isolate microglia from adult mice using this technique (Fig 4E). Quantitative PCR analysis of the isolated Cd11b^+^ fraction from the experiment demonstrates expression of the microglial markers *ITGAM*, *TLR4*, and *HEXB* and a near complete absence of astrocytic, oligodendrocyte, and neuronal marker genes (n = 3, p < 0.05).

### Magnetic sorting results in retention of processes

For these experiments, we employed transgenic rats expressing enhanced green fluorescent protein (eGFP) under the S100β promoter [23, 28]. S100β is a soluble calcium binding protein expressed in astrocytes [13, 29]. After cortical dissociation, the homogenate was split into two equal parts, with one half sent for FAC sorting. In the second half, we utilized the rabbit-anti-GLT-1 antibody and anti-rabbit microbeads as described in Fig 4. We first verified that the GLT-1 antibody was indeed able to isolate astrocytes across species via quantitative PCR analysis. Genes *SLC1A2* and *GFAP* were used to identify astrocytes, *MBP* was used to identify oligodendrocytes, *ITGAM* was used to identify microglia, and *MAP2* was used to identify neuronal populations. As seen in Fig 5A, qPCR data demonstrates isolation of astrocytes, with enriched expression of astrocyte genes and concomitant low expression of neuronal, microglia, and oligodendrocyte genes.

Morphological examination of eGFP^+^ astrocytes demonstrated that magnetically isolated cells maintained a complex morphology or ‘bushy’ appearance similar to that of the pre-sorted cells (Fig 5Bi and 5Bii). For comparison, we also examined eGFP+ astrocytes that had been FAC sorted. FAC sorted eGFP^+^ astrocytes were circular and had few, if any, attached processes (Fig 5Biii). Using Hoechst3342^+^ as a nuclear marker, we confirmed that pre-sorted cells, magnetically isolated eGFP+ astrocytes, and FAC sorted eGFP+ astrocytes were indeed intact cell somas with respective nuclei, rather than simply cellular debris. Utilizing eGFP expression, we were able to quantifiy the complexity of the magnetically isolated and FAC sorted astrocytes. Astrocytic eGFP is soluble, allowing for an accurate representation of cell shape and complexity. Here we used the Shape Index, defined as $\frac{{\text{Perimeter}}^{2}}{\text{area}}-4\pi$, to determine cellular complexity, with more complex cells exhibiting a higher index [25]. We found that magnetically sorted cells exhibited a statistically significantly higher index than FAC sorted cells (p < .001) (Fig 5C).

### Magnetic sorting allows for protein extraction

**Fig 6:**
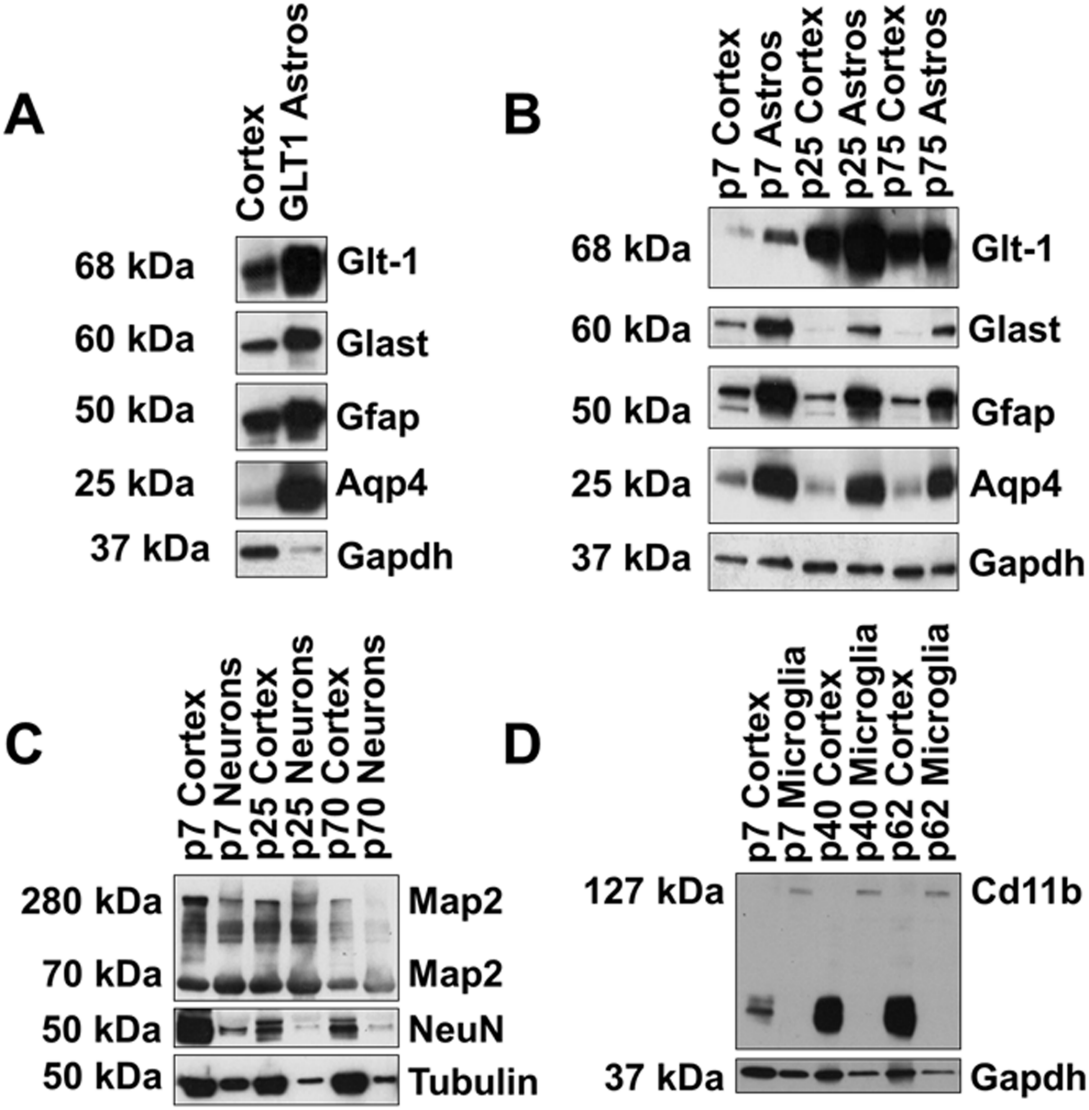
Protein expression in isolated populations. Following isolation, proteins were extracted and immunoblotting used to determine protein expression relative to whole cortex homogenates. A) Western blot analysis of proteins from magnetically enriched astrocyte populations in rat cortex demonstrates not only an increase in astrocytic protein expression, but also an increase in extrasomatic protein expression. B) Western blot analysis of proteins from magnetically separated cortical astrocyte populations in young, juvenile, and adult mice demonstrates similar trends in protein expression. Astrocyte-specific protein expression was enriched in the separated populations. For each time point, only one example is shown. C) Western blot analysis of magnetically separated neuronal populations in mice across developmental time points. Neuronal-specific protein expression was observed in the separated populations. D) Western blot analysis of microglia enriched populations following magnetic separations across development. Enrichment of microglia protein expression was observed, concomitant with depletion of non-specific primary antibody. For each time point, one experiment is shown.

Our next set of experiments were designed to determine if magnetic sorting would allow for the evaluation of protein expression, and more specifically if this technique was amenable for the study of extrasomatic proteins given the results in the previous experiment. For these experiments, an eGFP+ rat cortex (P25) was dissociated, and astrocytes were enriched using the GLT-1 antibody and magnetic bead sorting described above. Following enrichment, proteins were extracted and immunoblotting used to determine relative protein expression. A representative Western blot, shown in Fig 6A, demonstrates a marked increase in astrocyte protein expression in magnetic antibody enriched astrocytes relative to whole cortical homogenate, including higher levels of extrasomatic membrane-targeted proteins such as GLT-1 and GLAST. In addition, Aq4—which is expressed on astrocytic end feet—is also elevated in astrocytes isolated with magnetic beads relative to that of whole brain homogenate (Fig 6A).

We designed the next set of experiments to determine if proteins could be extracted from enriched populations in mouse cortex across development. In these experiments, the anti-ACSA-2 microbeads, Neuronal Isolation Kit, and Cd11b+ MicroBeads were utilized. As before, dissociated cells from whole cortex were incubated with anti-ACSA-2 microbeads and bound cells retained on the column. For neuronal separations, dissociated cells were incubated with an array of antibodies and unwanted cells remained bound to the column. For microglia enrichments, Cd11b+ microbeads were added to dissociated cells. Labeled cells were retained on the column. Following sorting, proteins were extracted and immunoblotting used to determine relative protein expression. One independent experiment is shown for all developmental time points. BCA protein assay data indicated that proteins were extracted from young (p7-p10), juvenile (p20-p30), and adult (p60-p75) animals in all cellular populations. Similar to the previous experiment, we found that within the isolated populations, there was a marked enrichment of astrocytic membrane-targeted proteins, including Glast, Glt-1, and Aq4, as well as GFAP (Fig 6B). In addition, we found that enriched neuronal populations exhibited expression of the neuronal markers NeuN and Map2 throughout development (Fig 6C). Within microglia populations, we observed enrichment of microglia marker Cd11b (Fig 6D). Interestingly, the enriched microglia populations also demonstrated a depletion of non-specific binding associated with the cd11b antibody that was observed in the cortex samples.

## Discussion

In the current study, we demonstrate that magnetic separation is a powerful technique for isolating major cell types within the central nervous system. This technique is efficient at isolating cells at different developmental time points and is amendable to customization using commercially available membrane-targeted antibodies, allowing for cell-specific isolation across development and animal species. Using this technique, we were able to isolate sufficient quantities of RNA for downstream applications, including quantitative PCR and RNA sequencing. Magnetic separation also allows for cellular enrichment from relatively small tissue samples, as we found that one-half of a p7 cortex was sufficient to isolate cell populations and still obtain RNA of quality and quantity for downstream applications. This technique is relatively gentle. Astrocytes retained a dense network of processes, enabling analysis of extrasomatic proteins. Magnetic cell separation is a low cost process that does not require specialized training or equipment, and eliminates the need for fluorescent cells. We propose that this technique will add significantly to our ability to efficiently study isolated/enriched cell populations in the CNS.

The ability to study single cell populations in isolation is an important next step in our understanding of specific cellular and molecular processes. This task is straightforward when subsets of genes that are specific to a certain cell population are assayed, but is not possible in a homogenate of multiple cell types where significant overlap in gene expression exists [13, 14]. Enrichment of the cellular population of interest permits a more accurate portrait of cell-type specific expression by removing dilution effects seen in whole homogenate [30, 31]. For example, when examining off target CNS effects of statins, Dong et al. identified cell type specific differential expression of genes associated with neurodegeneration [32]. Consequently, separation of cell types advances the understanding of how distinct and overlapping expression contributes to not only specific cellular function, but also general nervous system function. This notion has proved particularly intriguing in the neurodevelopmental disorder Rett syndrome, which is caused by mutations in the ubiquitously expressed transcriptional regulator methyl CpG binding protein 2 (MeCP2). Here, global restoration of MeCP2, as well as neuronal specific expression and astrocyte specific expression, rescues overt disease phenotypes [2, 33–36]. A better understanding of cell-autonomous and non-autonomous effects on gene regulation will aid in our understanding of such phenomena. Magnetic cellular separations provide a powerful option in elucidating cell-specific and non-cell autonomous disease mechanisms.

To date, the use of magnetic beads to separate cellular populations has been limited to very young animals, and more specifically, has largely been utilized for the purification of cells during early post-natal development (<p7) for use in culture systems [18, 20, 37, 38]. In fact, a literature search found only three papers demonstrating the usage of this technique to directly study *in vivo* central nervous system processes [7, 39, 40]. Ohtsuki et al found that capillary endothelial cells isolated from adult mice express mRNA specific for tight junctions in mouse brain. Microglia isolated in the same manner were found to have phenotypic characteristics similar to those *in vivo*, such as TNF-α production [40]. In addition, astrocytes and neurons were sequentially isolated from postnatal day 9 CD1 mice for ex vivo analysis [7].

Using an ACSA-2 antibody, we isolated astrocytes from early postnatal development into adulthood. We found that our enriched population also expressed genes that have previously been found to be astrocyte-specific markers, while concomitantly demonstrating low expression of non-astrocytic markers [4, 13, 14]. We also enriched for neuronal populations using a negative selection method. Similar to the astrocytic isolations, our enriched neuronal population expressed neuron-specific genes with little to no expression of other cellular markers, such as Gfap, Cd11b, and Mbp [4, 13, 14]. Given the efficiency of separation, magnetic cell separation is underutilized for separating distinct cell CNS cell populations in adult mice and rats.

Researcher customization and the lack of a requirement for cell-specific fluorescent reporters adds to the appeal of magnetic based cell separation. Using an antibody specific for an extracellular epitope of glutamate transporter 1 (GLT-1), we were able to isolate astrocytes across development in mice. This antibody also shares 100% sequence homology with rats and humans. Using this antibody, we were also able to isolate RNA and protein from GLT-1^+^/GFAP^+^ astrocytes from rat cortex. This technique may be of particular interest to researchers breeding multiple lines of transgenic mice that do not have fluorescent reporters for the cell of interest, or alternatively when using animal models in which the generation of cell specific reporters is not feasible. While customization is an advantage to this technique, there are a few caveats. Antibodies must be specific to an extracellular portion of the protein of interest and variation may exist in different lots of antibodies. Researchers must take care to choose appropriate antibodies. This technique is also amenable to isolating subsets of cells, assuming appropriate antibodies are available.

Our data indicate astrocytes isolated using the magnetic separations are significantly more morphologically complex than those isolated by FAC sorting, suggesting that magnetic isolation of cells is gentler than FAC sorting. This is particularly important in the CNS where arborizations and distal processes demonstrate protein signatures that are distinct from the cell soma. For instance, axon terminals contain the molecular machinery for neurotransmitter release, while astrocytes express high levels of machinery for ionic homeostasis at terminals of distal processes [41–44]. Loss of cellular processes during FAC sorting represents a major limitation in the utilization of the technique in proteomic studies, while highlighting the utility of magnetic isolation.

Magnetic based cell isolation kits for isolating neurons are designed for use at very young postnatal ages, before myelination begins. In p7-p10 cortex, we isolated neurons with little contamination from other cell types; however, isolation of neurons from older animals resulted in significant contamination, as evidenced by oligodendrocyte gene expression. As seen in Fig 3A–3C, a pure neuronal population was enriched after removal of myelin, specifically at older time points. The neuronal isolation technique is based on a negative selection, which may have contributed to oligodendrocyte contamination. Using this technique, we found that expression of neuronal markers remained in the unsorted population. Further optimization is required to capture the entire neuronal population. As mentioned above, the isolation of specific neuronal populations is only limited by available antibodies.

While not a novel technique, this powerful method is highly underutilized. We found magnetic cell sorting efficient at isolating astrocytes, neurons, and microglia from a cortical homogenate. In fact, using a positive selection method, astrocyte markers were nearly completely depleted from cortical homogenates (Fig 2D, 2E and 2G). Magnetic sorting is relatively fast—from dissociation to selection of cell populations, magnetic sorting took an average of 1–2 hours with little training or specialized equipment. A simple cost evaluation of supplies and reagents for the magnetic sorting indicated a relatively low cost per sample isolation. We propose that magnetic soring will prove to be a highly useful technique for the examination of CNS cell specific gene and protein expression.

## Supporting Information

S1 DataSet Quantitative PCR and Shape Index data. (ZIP)

S1 Fig FAC sorting of eGFP positive astrocytes. Astrocytes were gated based on forward and side scatter plots as previously described[23, 24]. **A.** An ethidium bromide based dead cell indicator was utilized to gate for a live cell population (P2), **B,C**, The excluded P1, ETBr+ population demonstrates high side scatter, while the P2 ETBr- fraction demonstrates lower side scatter. **D**. The live cell population (P2) demonstrated two populations—one with a high eGFP profile (blue) and a low eGFP profile (green). Fluorescent microscope visual examination of the populations following incubation with Hoechst demonstrated that the low eGFP population lacked co-localization of eGFP and Hoechst. Furthermore this population appeared significantly smaller than the high eGFP population, which exhibited co-localization with Hoechst, indicating the low eGFP population likely represented astrocyte cellular debris.
(TIF)

S1 Table MicroBead kits and antibodies used for isolating CNS populations. (DOCX)

S2 Table RNA Integrity Numbers for whole cortex homogenate and isolated cell populations. (DOCX)
