# Supplementary figures and images for: Novel Applications of Magnetic Cell Sorting to Analyze Cell-Type Specific Gene and Protein Expression in the Central Nervous System
Source: PLoS One. 2016 Feb 26;11(2):e0150290. doi: 10.1371/journal.pone.0150290 (PMC4769085; doi:10.1371/journal.pone.0150290)

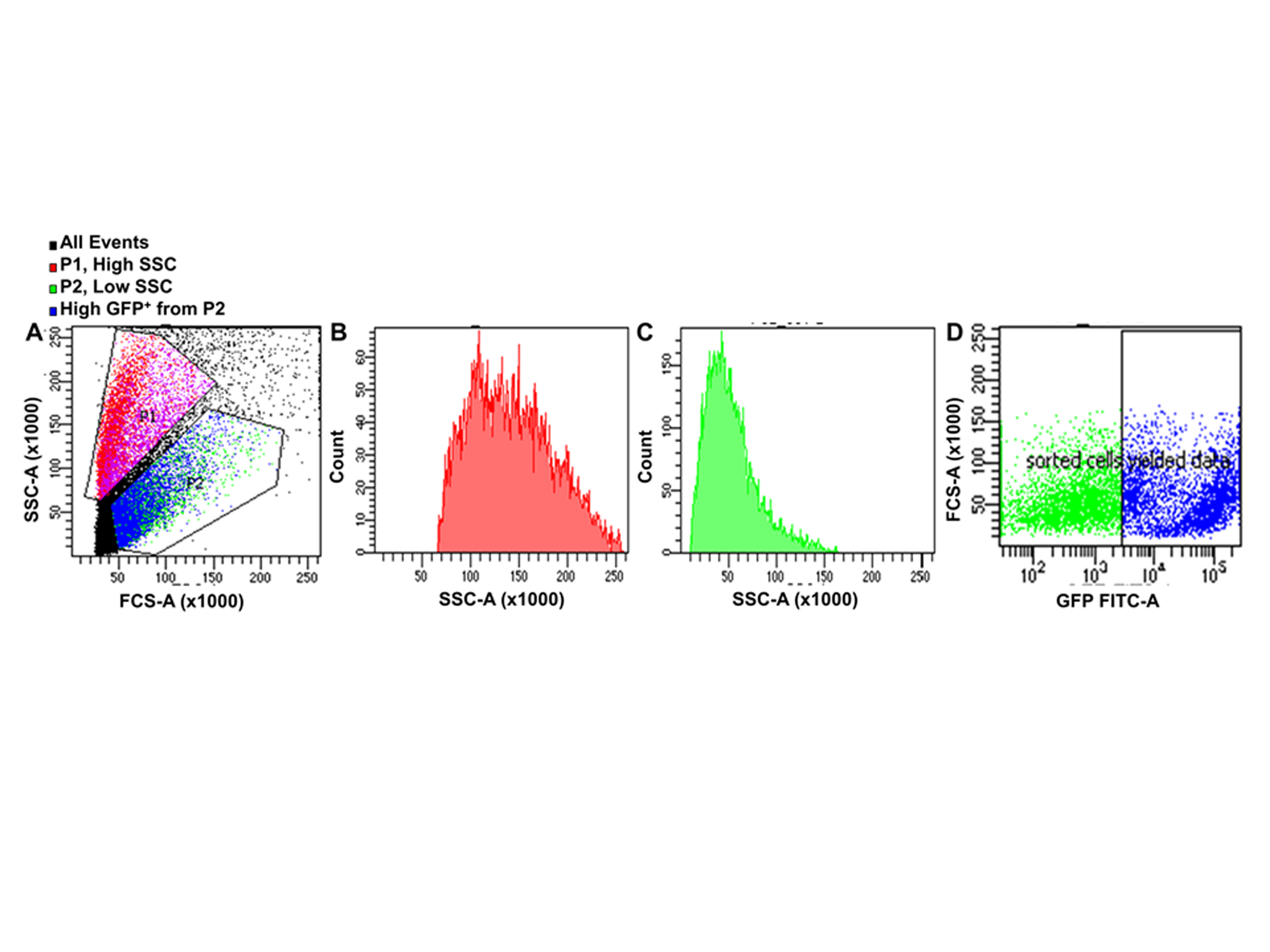

Supplement: S1 Fig — Astrocytes were gated based on forward and side scatter plots as previously described[23, 24]. A. An ethidium bromide based dead cell indicator was utilized to gate for a live cell population (P2), B,C, The excluded P1, ETBr+ population demonstrates high side scatter, while the P2 ETBr- fraction demonstrates lower side scatter. D. The live cell population (P2) demonstrated two populations—one with a high eGFP profile (blue) and a low eGFP profile (green). Fluorescent microscope visual examination of the populations following incubation with Hoechst demonstrated that the low eGFP population lacked co-localization of eGFP and Hoechst. Furthermore this population appeared significantly smaller than the high eGFP population, which exhibited co-localization with Hoechst, indicating the low eGFP population likely represented astrocyte cellular debris. (TIF) [file pone.0150290.s002.tif]
