# Supplementary material for: Novel Applications of Magnetic Cell Sorting to Analyze Cell-Type Specific Gene and Protein Expression in the Central Nervous System
Source: PLoS One. 2016 Feb 26;11(2):e0150290. doi: 10.1371/journal.pone.0150290 (PMC4769085; doi:10.1371/journal.pone.0150290)
Supplement: S1 Table — (DOCX) [file pone.0150290.s003.docx]

| **Material Used** | **Company** | **Catalog Number** |
| --- | --- | --- |
| Anti-ACSA-2 MicroBead Kit | Miltenyi Biotec | 130-097-678 |
| Anti-Rabbit IgG MicroBeads | Miltenyi Biotec | 130-048-602 |
| CD11b (Microlia) Microbeads | Miltenyi Biotec | 130-093-634 |
| Glt1 (extracellular) antibody | Alomone Labs | ACG-022 |
| LD Columns | Miltenyi Biotec | 130-042-901 |
| LS Columns | Miltenyi Biotec | 130-042-401 |
| Myelin Removal Beads II | Miltenyi Biotec | 130-093-634 |
| Neuron Isolation Kit | Miltenyi Biotec | 130-098-752 |
| Papain Dissociation Kit | Worthington Biochemical | LK003153 |
