# Supplementary material for: Novel Applications of Magnetic Cell Sorting to Analyze Cell-Type Specific Gene and Protein Expression in the Central Nervous System
Source: PLoS One. 2016 Feb 26;11(2):e0150290. doi: 10.1371/journal.pone.0150290 (PMC4769085; doi:10.1371/journal.pone.0150290)
Supplement: S2 Table — (DOCX) [file pone.0150290.s004.docx]

| **Cellular Population** | **RNA Integrity Number (RIN)** |
| --- | --- |
| Cortex | 7.7 +/- .67 |
| ACSA-2 Astrocytes | 7.76 +/- .09 |
| GLT-1 Astrocytes | 7.75 +/- .12 |
| Neurons | 6.27 +/- .44 |
| Microglia | 6.8 +/- .12 |
